# Four Cases of Suspected Levetiracetam-Induced Asymptomatic Rhabdomyolysis

**DOI:** 10.7759/cureus.41666

**Published:** 2023-07-10

**Authors:** Satoshi Saito, Mutsumi Iijima, Ryotaro Ikeguchi, Kentaro Ishizuka, Kazuo Kitagawa

**Affiliations:** 1 Department of Neurology, Tokyo Women’s Medical University School of Medicine, Tokyo, JPN

**Keywords:** epilepsy, serum creatine kinase, rhabdomyolysis, levetiracetam, antiepileptic drug

## Abstract

Rhabdomyolysis is a known side effect of levetiracetam. In general, a patient with rhabdomyolysis complains of muscle pain and swelling. Herein, we report four cases of asymptomatic levetiracetam-induced rhabdomyolysis. In all the four cases, the seizures resolved after more than five days. The patients received continuous fluid replacement from the time they were admitted to our hospital. However, serum creatine kinase (CK) levels continued to rise without symptoms consistent with rhabdomyolysis. The serum CK level improved rapidly when levetiracetam was replaced with lacosamide. Because levetiracetam occasionally causes asymptomatic rhabdomyolysis, routine blood tests should be performed after its initiation.

## Introduction

Levetiracetam, a drug used for treating focal seizures [1], is also used as adjunctive therapy for myoclonic seizures [2] as well as primary, generalized, tonic-clonic seizures [3]. The drug has a few adverse effects [4]. Some of the side effects include somnolence (14%), headache (10%), fatigue (8%), injury by accident (8%), and dizziness (7%) [5]. In Japan, the Pharmaceuticals and Medical Devices Agency reported cases of rhabdomyolysis between 2013 and 2015. The pharmaceutical manufacturing company recommends the discontinuation of levetiracetam if patients experience muscle pain, weakness, and elevations in serum creatine kinase (CK) and myoglobin levels. To date, the pathogenesis of levetiracetam-induced rhabdomyolysis remains unclear. In general, a patient with rhabdomyolysis complains of muscle pain and swelling; however, 50% of the patients with rhabdomyolysis do not report muscle pain. Therefore, it could be difficult to diagnose rhabdomyolysis in the initial clinical evaluation based only on muscular symptoms and signs [6]. To the best of our knowledge, this is the first case report of levetiracetam-induced asymptomatic rhabdomyolysis, with four other similar cases reported previously [7-10].

## Case presentation

Case 1

**Figure 1:**
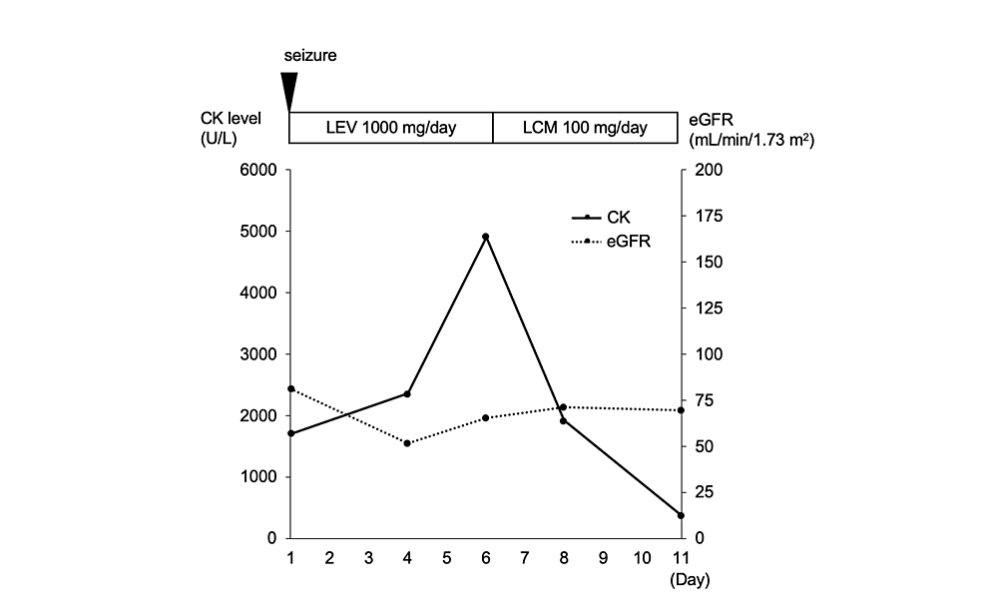
Clinical course of case 1. The solid line shows serum CK levels, and the dotted line shows eGFRs. Serum CK levels decreased after switching from LEV to LCM. CK, creatine kinase; eGFR, estimated glomerular filtration rate; LCM, lacosamide; LEV, levetiracetam

A 34-year-old man had started taking an antiepileptic drug (AED) at the age of 12 and discontinued it a few years later. At the age of 34, he had an episode of impaired awareness and generalized tonic-clonic seizure while working. The generalized tonic-clonic seizure lasted approximately 10 seconds. The results of the blood test performed on admission were as follows: CK level of 1,703 U/L, aspartate aminotransferase (AST) level of 55 U/L, alanine transaminase (ALT) level of 41 U/L, lactate dehydrogenase (LDH) level of 400 U/L, Cr level of 0.88 mg/dL, and estimated glomerular filtration rate (eGFR) level of 81.1 mL/min/1.73 m^2^. Electroencephalography (EEG) revealed sharp waves in the right anterior temporal lesion. Magnetic resonance imaging (MRI) fluid-attenuated inversion recovery (FLAIR) revealed a high signal and atrophy in the right hippocampus. Based on the EEG and MRI FLAIR findings, temporal lobe epilepsy with hippocampal sclerosis was diagnosed, and he was placed on levetiracetam at a dose of 1,000 mg/day. Continuous intravenous fluid replacement was also initiated to protect his renal function from rhabdomyolysis until discharge. On day 4 of hospitalization, his serum CK level increased from 1,703 U/L to 2,357 U/L, despite the absence of seizures and renal dysfunction. He had not commenced any new medication other than levetiracetam since his admission. On day 6, his serum CK level increased to 4,904 U/L. We suspected that the high CK level was a side effect of levetiracetam; hence, we changed the medication to lacosamide. On day 8 of hospitalization, the serum CK level rapidly decreased (Figure 1).

Case 2

**Figure 2:**
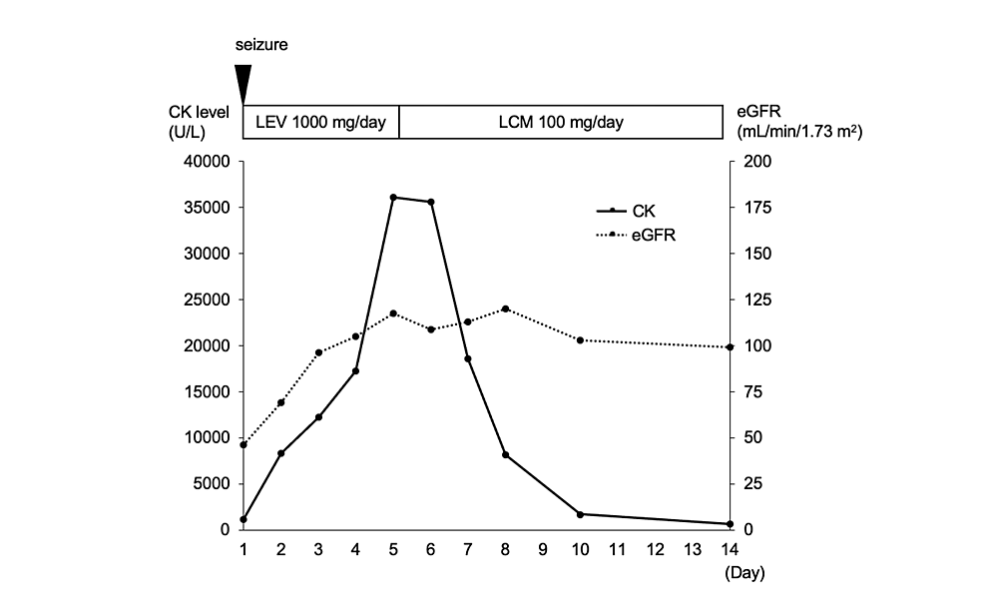
Clinical course of case 2. The solid line shows serum CK levels, and the dotted line shows eGFRs. Serum CK levels decreased after switching from LEV to LCM. CK, creatine kinase; eGFR, estimated glomerular filtration rate; LCM, lacosamide; LEV, levetiracetam

A 60-year-old man had two episodes of generalized tonic-clonic seizures and a history of alcoholism. He was transferred to our hospital because of a generalized tonic-clonic seizure and impairment of consciousness. The generalized tonic-clonic seizure lasted 10 minutes. The results of the blood tests performed on admission were as follows: CK level of 1,117 U/L, AST level of 35 U/L, ALT level of 55 U/L, LDH level of 332 U/L, Cr level of 1.27 mg/dL, and eGFR level of 46.1 mL/min/1.73 m^2^. We established a diagnosis of status epilepticus and started treatment with levetiracetam (1,000 mg/day) and midazolam. On day 2 of hospitalization, his consciousness improved. His EEG and MRI were normal. Continuous intravenous fluid replacement was performed to protect renal function from rhabdomyolysis until he was discharged. On day 5, his serum CK level increased from 1,117 U/L to 36,060 U/L despite improvements in renal dysfunction. No seizures were observed from day 2 to day 5. He had no muscle pain or weakness and had not started any new medication that caused rhabdomyolysis other than levetiracetam. We suspected an adverse reaction to levetiracetam and changed the medication to lacosamide. His serum CK level decreased to 35,560 U/L on day 6, 18,559 U/L on day 7, and 8,130 U/L on day 8. The serum CK level decreased promptly after the medication was changed from levetiracetam to lacosamide (Figure 2).

Case 3

**Figure 3:**
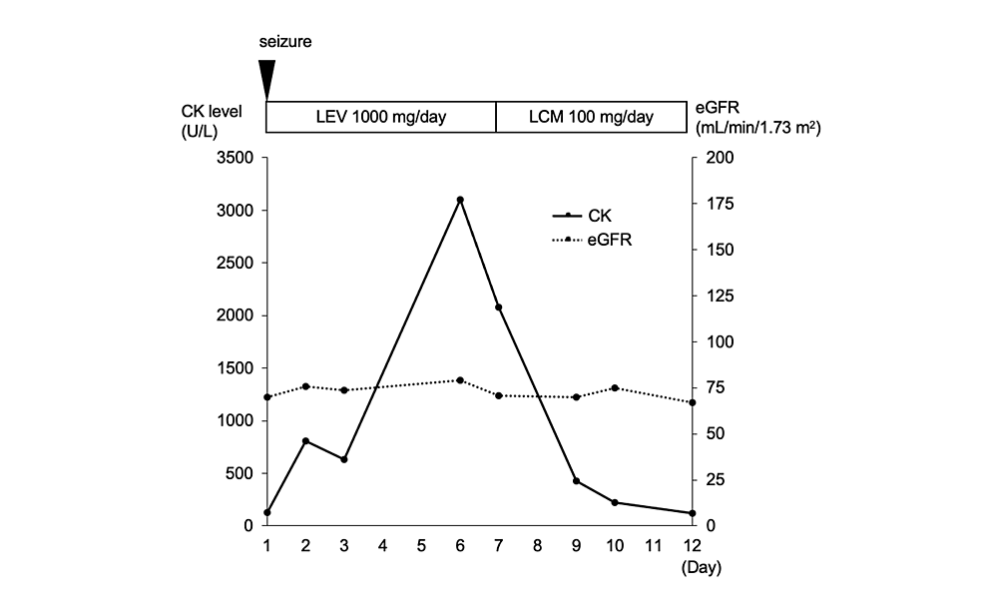
Clinical course of case 3. The solid line shows serum CK levels, and the dotted line shows eGFRs. The improvement in serum CK levels was maintained after switching from LEV to LCM CK, creatine kinase; eGFR, estimated glomerular filtration rate; LCM, lacosamide; LEV, levetiracetam

A 25-year-old woman who had no seizures before admission and had undergone surgery for a pulmonary arteriovenous fistula at the age of 19 years was transferred to our hospital because of a generalized tonic-clonic seizure. The generalized tonic-clonic seizure lasted 30 minutes. The results of the blood tests performed on admission were as follows: CK level of 128 U/L, AST level of 18 U/L, ALT level of 12 U/L, LDH level of 182 U/L, Cr level of 0.83 mg/dL, and eGFR level of 69.8 mL/min/1.73 m^2^. Brain MRI revealed a 16-mm abscess in the left temporal lobe. We started treatment with levetiracetam (1,000 mg/day), meropenem, and vancomycin. On day 2, her level of consciousness improved. Continuous intravenous fluid replacement was performed to protect renal function from rhabdomyolysis until she was discharged. On day 3, her serum CK level decreased from 806 U/L to 632 U/L. On day 6, the serum CK level increased again to 3,101 U/L despite the absence of renal dysfunction. No seizures were observed from day 2 to day 6. She had no muscle pain or weakness. On day 7, her serum CK level was 2,078 U/L. On day 14, meropenem was de-escalated to ceftriaxone. She had not started any new medication that could induce rhabdomyolysis other than levetiracetam and meropenem. We suspected levetiracetam side effects and switched from levetiracetam to lacosamide. After the change, the serum CK level did not increase again (Figure 3).

Case 4

**Figure 4:**
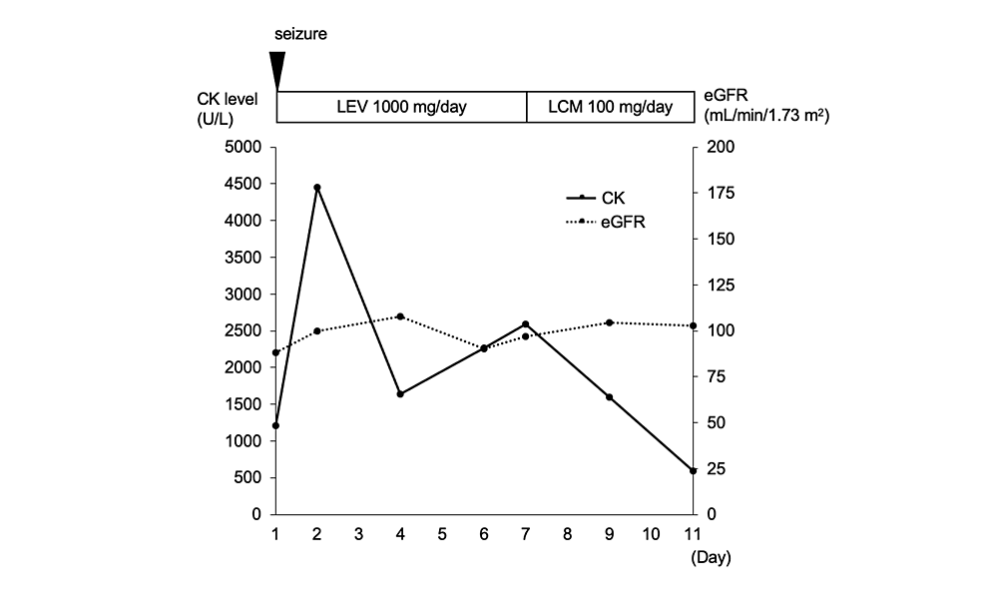
Clinical course of case 4. The solid line shows serum CK levels, and the dotted line shows eGFRs. Serum CK levels decreased after switching from LEV to LCM. CK, creatine kinase; eGFR, estimated glomerular filtration rate; LCM, lacosamide; LEV, levetiracetam

A 32-year-old man had autism and no AEDs before admission. At the age of 12 years, he had generalized tonic-clonic seizures. At the age of 32 years, he was transferred to our hospital because of a generalized tonic-clonic seizure. The generalized tonic-clonic seizure lasted approximately 10 seconds. The results of the blood tests performed on admission were as follows: CK level of 1,211 U/L, AST level of 31 U/L, ALT level of 26 U/L, LDH level of 276 U/L, Cr level of 0.83 mg/dL, and eGFR level of 88 mL/min/1.73 m^2^. His EEG and MRI were normal. We started treatment with levetiracetam (1,000 mg/day). On day 2, his level of consciousness improved. His serum CK peaked at 4,455 U/L. Continuous intravenous fluid replacement was performed to protect renal function from rhabdomyolysis until he was discharged. On day 4, his serum CK level decreased to 1,639 U/L. On day 6, the serum CK level increased again to 2,261 U/L despite the absence of renal dysfunction. No seizures were observed from day 2 to day 6. He had no muscle pain or weakness. On day 7, his serum CK level further increased to 2,593 U/L. He had not started any new medication that could induce rhabdomyolysis other than levetiracetam. Because of the suspected side effects, we switched from levetiracetam to lacosamide. After the change, the serum CK level did not increase again (Figure 4).

## Discussion

The pathogenesis of levetiracetam-induced rhabdomyolysis is still unclear. Levetiracetam acts upon by binding to synaptic vesicle protein 2A (SV2A), the binding site of levetiracetam in the brain, and modulating the function of SV2A. The mechanism of action of levetiracetam is different from that of other AEDs [11]. SV2A is selectively present on the motor nerve endings of the slow muscle fibers of the mouse [12]. Levetiracetam affects specific patients with high sensitivity of the SV2A protein in motor nerve terminals, possibly causing rhabdomyolysis. When rhabdomyolysis occurs, myoglobin, CK, and potassium are released into the blood and cause electrolyte abnormalities and renal dysfunction [13]. Constant monitoring of the serum CK level is important for the detection of asymptomatic rhabdomyolysis and prevention of renal function impairments.

In a previous study, rhabdomyolysis developed 1-15 days after the administration of levetiracetam [14]. Serum CK levels rise substantially following a generalized tonic-clonic seizure, usually between 24 and 48 hours after the seizure [15]. In our cases, serum CK was elevated over five days. Indeed, in a few cases, the CK level remained high over four days; however, this is rare [16]. We performed continuous intravenous fluid replacement to protect renal function from rhabdomyolysis until the patient was discharged. Thus, the patient’s renal function did not deteriorate. Therefore, the changes in the serum CK levels could not be explained by the postictal period alone. Serum CK levels improved promptly after switching from levetiracetam to lacosamide. The high serum CK levels were considered a side effect of levetiracetam. In cases 3 and 4, the serum CK level peaked and began to rise again before the treatment of these patients was switched to lacosamide, despite the continuous fluid replacement. Therefore, the increased serum CK level could not be explained by the seizure alone. Because the serum CK level did not increase again after switching to lacosamide, we concluded that these phenomena were adverse effects of levetiracetam. In case 4, meropenem was used to treat brain abscess. Although it has been reported to cause antibiotic-related rhabdomyolysis [17], meropenem did not cause rhabdomyolysis in this case because the serum CK level had already improved when meropenem was de-escalated to ceftriaxone.

## Conclusions

Rhabdomyolysis is a condition that causes muscle pain and swelling. However, in up to 50% of the cases, it may occur without muscle pain. This condition could occur as an adverse effect of levetiracetam, an anticonvulsive medication. We present the cases of four patients with seizures who experienced rhabdomyolysis as an adverse effect of levetiracetam. The patients also received continuous fluid replacement therapy to protect their renal function from rhabdomyolysis. However, they developed rhabdomyolysis, as observed by increases in CK levels. Levetiracetam has the potential to cause asymptomatic rhabdomyolysis. Therefore, continuous monitoring of the serum CK levels is important to detect asymptomatic rhabdomyolysis.
